# Suppressing qubit dephasing using real-time Hamiltonian estimation

**DOI:** 10.1038/ncomms6156

**Published:** 2014-10-08

**Authors:** M. D. Shulman, S. P. Harvey, J. M. Nichol, S. D. Bartlett, A. C. Doherty, V. Umansky, A. Yacoby

**Affiliations:** 1 Department of Physics, Harvard University, Cambridge, Massachusetts 02138, USA; 2 Centre for Engineered Quantum Systems, School of Physics, The University of Sydney, Sydney, New South Wales 2006, Australia; 3 Braun Center for Submicron Research, Department of Condensed Matter Physics, Weizmann Institute of Science, Rehovot 76100, Israel

## Abstract

Unwanted interaction between a quantum system and its fluctuating environment leads to decoherence and is the primary obstacle to establishing a scalable quantum information processing architecture. Strategies such as environmental and materials engineering, quantum error correction and dynamical decoupling can mitigate decoherence, but generally increase experimental complexity. Here we improve coherence in a qubit using real-time Hamiltonian parameter estimation. Using a rapidly converging Bayesian approach, we precisely measure the splitting in a singlet-triplet spin qubit faster than the surrounding nuclear bath fluctuates. We continuously adjust qubit control parameters based on this information, thereby improving the inhomogenously broadened coherence time 
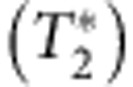
 from tens of nanoseconds to >2 μs. Because the technique demonstrated here is compatible with arbitrary qubit operations, it is a natural complement to quantum error correction and can be used to improve the performance of a wide variety of qubits in both meteorological and quantum information processing applications.

## Abstract (web summary)

Decoherence is anathema to quantum systems, as it reduces their performance and stability. Shulman *et al.* show that real-time Hamiltonian parameter estimation can significantly increase the coherence time of a qubit by enabling continuous adjustment of its control parameters.

## Graphical abstract

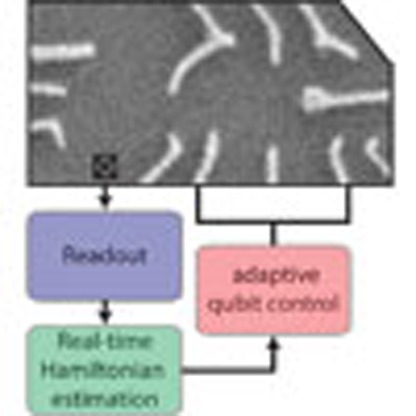

Hamiltonian parameter estimation is a rich field of active experimental and theoretical research that enables precise characterization and control of quantum systems^1^. For example, magnetometry schemes employing Hamiltonian learning have demonstrated dynamic range and sensitivities exceeding those of standard methods^2^,^3^. Such applications focused on estimating parameters that are quasistatic on experimental timescales. However, the effectiveness of Hamiltonian learning also offers exciting prospects for estimating fluctuating parameters responsible for decoherence in quantum systems.

The quantum system that we study is a singlet-triplet (*S*−*T*_0_) qubit^4^,^5^ which is formed by two gate-defined lateral quantum dots (QDs) in a GaAs/AlGaAs heterostructure (Fig. 1a, Supplementary Fig. 1), similar to that of refs 6, 7. The qubit can be rapidly initialized in the singlet state |*S*› in ≈20 ns and read out with 98% fidelity in ≈1 μs (refs 8, 9; Supplementary Fig. 2). Universal quantum control is provided by two distinct drives^10^: the exchange splitting, *J*, between |*S*› and |*T*_0_›, and the magnetic field gradient, Δ*B*_*z*_, due to the hyperfine interaction with host Ga and As nuclei. The Bloch sphere representation for this qubit can be seen in Fig. 1b. In this work, we focus on qubit evolution around Δ*B*_*z*_ (Fig. 2a). Due to statistical fluctuations of the nuclei, Δ*B*_*z*_ varies randomly in time, and consequently oscillations around this field gradient decay in a time 
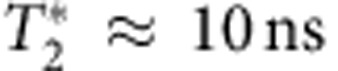
 (ref. 4). A nuclear feedback scheme relying on dynamic nuclear polarization^11^ can be employed to set the mean gradient, (g*μ_B_Δ*B*_*z*_/*h*≈60 MHz in this work) as well as reduce the variance of the fluctuations. Here, g*≈−0.44 is the effective gyromagnetic ratio in GaAs, *μ*_B_ is the Bohr magneton and *h* is Planck’s constant. In what follows, we adopt units where g*μ_B_/*h*=1. The nuclear feedback relies on the avoided crossing between the |*S*› and |*T*_+_› states. When the electrons are brought adiabatically through this crossing, their total spin changes by Δ*m*_s_=±1, which is accompanied by a nuclear spin flip to conserve angular momentum. With the use of this feedback, the coherence time improves to 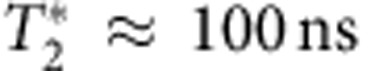 (ref. 11; Fig. 2b), limited by the low nuclear pumping efficiency^10^. Crucially, the residual fluctuations are considerably slower than the timescale of qubit operations^12^.

In this work we employ techniques from Hamiltonian estimation to prolong the coherence of a qubit by more than a factor of 30. Importantly, our estimation protocol, which is based on recent theoretical work^13^, requires relatively few measurements (≈100) which we perform rapidly enough (total time ≈100 μs) to resolve the qubit splitting faster than its characteristic fluctuation time. We adopt a paradigm in which we separate experiments into ‘estimation’ and ‘operation’ segments, and we use information from the former to optimize control parameters for the latter in real-time. Our method dramatically prolongs coherence without using complex pulse sequences such as those required for non-identity dynamically decoupled operations^14^.

## Results

### Rotating frame *S*−*T*_0_ qubit

To take advantage of the slow nuclear dynamics, we introduce a method that measures the fluctuations and manipulates the qubit on the basis of precise knowledge but not precise control of the environment. We operate the qubit in the rotating frame of Δ*B*_*z*_, where qubit rotations are driven by modulating *J* at the frequency 
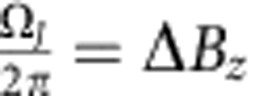
 (refs 15, 16). This is in contrast to traditional modes of operation of the *S*−*T*_0_ qubit, which rely on DC voltage pulses. To measure Rabi oscillations, the qubit is adiabatically prepared in the ground state of Δ*B*_*z*_ (|*ψ*›=|↑↓›), and an oscillating *J* is switched on (Fig. 2e), causing the qubit to precess around *J* in the rotating frame. In addition, we perform a Ramsey experiment (Fig. 2c) to determine 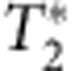, and as expected, we observe the same decay (Fig. 2d) as Fig. 2b. More precisely, the data in Fig. 2d represent the average of 1,024 experimental repetitions of the same qubit operation sequence immediately following nuclear feedback. The feedback cycle resets Δ*B*_*z*_ to its mean value (60 MHz) with residual fluctuations of 
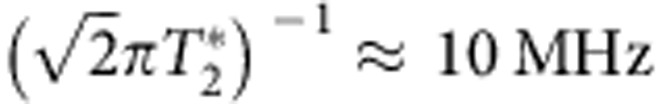
 between experimental repetitions. However, within a given experimental repetition, Δ*B*_*z*_ is approximately constant. Therefore we present an adaptive control scheme where, following nuclear feedback, we quickly estimate Δ*B*_*z*_ and tune 
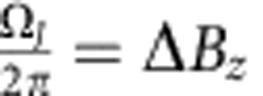
 to prolong qubit coherence (Fig. 3a).

### Bayesian estimation

To estimate Δ*B*_*z*_ , we repeatedly perform a series of single-shot measurements after allowing the qubit to evolve around Δ*B*_*z*_ (using DC pulses) for some amount of time (Fig. 2a). Rather than fixing this evolution time to be constant for all trials, we make use of recent theoretical results in Hamiltonian parameter estimation^13^,^16^,^17^ and choose linearly increasing evolution times, *t*_*k*_=*kt*_samp_, where *k*=1,2,…*N*. We choose the sampling time *t*_samp_ such that the estimation bandwidth 
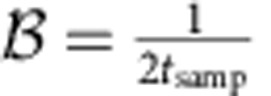
 is several times larger than the magnitude of the residual fluctuations in Δ*B*_*z*_, roughly 10 MHz. With a Bayesian approach to estimate Δ*B*_*z*_ in real-time, the longer evolution times (large *k*) leverage the increased precision obtained from earlier measurements to provide improved sensitivity, allowing the estimate to outperform the standard limit associated with repeating measurements at a single evolution time. Denoting the outcome of the *k*th measurement as *m*_*k*_ (either |*S*› or |*T*_0_›), we define *P*(*m*_*k*_|Δ*B*_*z*_) as the conditional probability for *m*_*k*_ given a value Δ*B*_*z*_. We write


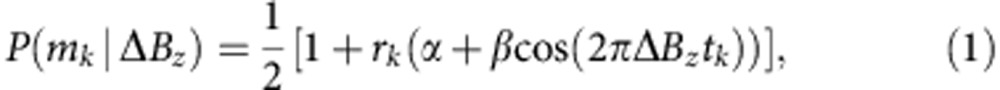


where *r*_*k*_=1 (−1) for *m*_*k*_=|*S*›(|*T*_0_›), and *α*=0.25 and *β*=0.67 are parameters determined by the measurement error and axis of rotation on the Bloch sphere (see Methods). Since we assume that earlier measurement outcomes do not affect later ones (that is, that there is no measurement back-action), we write the conditional probability for Δ*B*_*z*_ given the results of *N* measurements as:


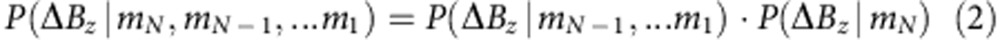



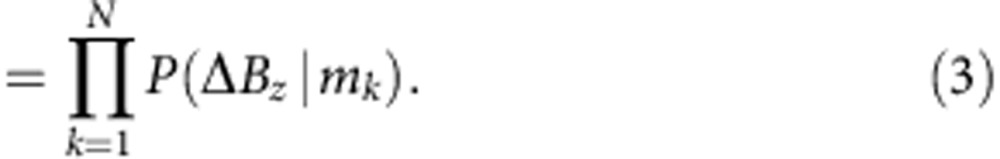


Using Bayes’ rule, that is, *P*(Δ*B*_*z*_|*m*_*k*_)=*P*(*m*_*k*_|Δ*B*_*z*_)*P*(Δ*B*_*z*_)/*P*(*m*_*k*_), and equation (1), we can rewrite equation (3) as:


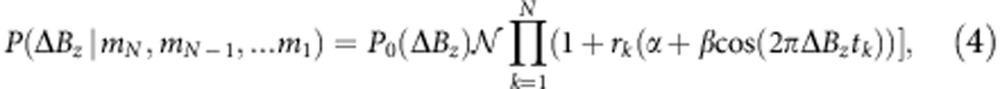


where *N* is a normalization constant and *P*_0_(Δ*B*_*z*_) is a prior distribution to which the algorithm is empirically insensitive, and which we take to be a constant over the estimation bandwidth. After the last measurement, we find the value of Δ*B*_*z*_ that maximizes the posterior distribution *P*(Δ*B*_*z*_|*m*_*N*_,*m*_*N*−1_,...*m*_1_).

### Adaptive control

**Figure 1:**
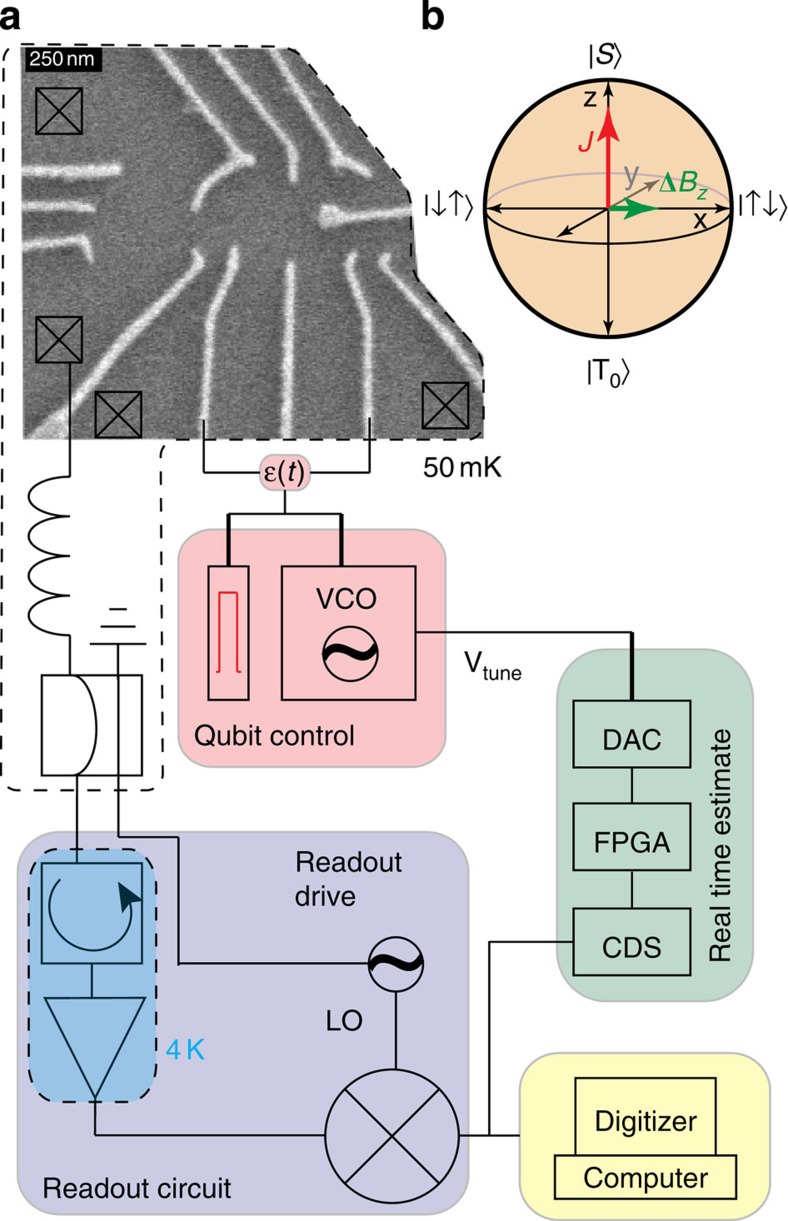
Experimental apparatus. (**a**) A scanning electron microscope image of the double QD with a schematic of the apparatus used for adaptive qubit control. A floating metal gate protruding from the right can be seen which increases the capacitance between the qubit and an adjacent qubit (not pictured), which is left inactive for this work. The reflected read-out drive signal is demodulated to DC, digitized by a correlated double sampler (CDS), and Δ*B*_*z*_ is estimated in real time by the field-programmable gate array (FPGA). The FPGA updates the digital to analogue converter (DAC) to keep the voltage controlled oscillator (VCO) resonant with the estimated value of Δ*B*_*z*_. The VCO controls the voltage detuning, *ε*(*t*) between the QDs, which, in turn, modulates *J* at Ω_*J*_. (**b**) The Bloch sphere representation for the *S*−*T*_0_ qubit showing the two axes of control, *J* and Δ*B*_*z*_.

**Figure 2:**
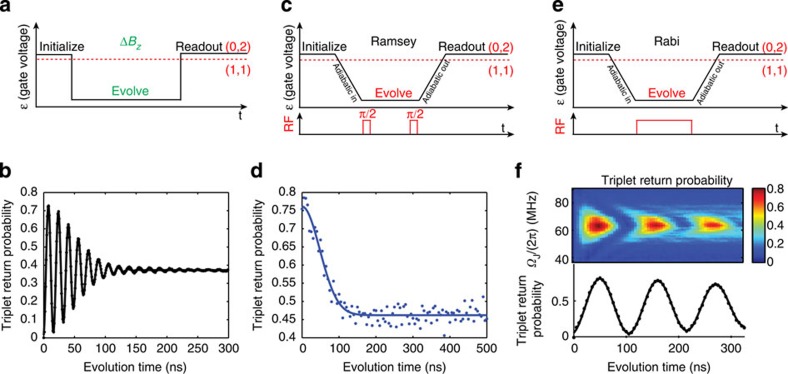
Δ*B*_*z*_ oscillations. (**a**) The pulse sequence used to estimate Δ*B*_*z*_. (**b**) Using nuclear feedback, Δ*B*_*z*_ oscillations decay in a coherence time 
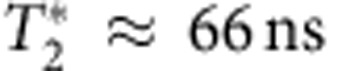
 due to residual slow fluctuations in Δ*B*_*z*_. (**c**) The Ramsey sequence used to operate the *S*-*T*_0_ qubit in the rotating frame. (**d**) The Ramsey contrast (blue dots) decays in a characteristic time (solid line fit 
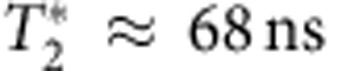
) similar to the oscillations in **b** due to the same residual slow fluctuations in Δ*B*_*z*_. (**e**) The Rabi pulse sequence used to drive the qubit in the rotating frame. (**f**) The rotating frame *S*−*T*_0_ qubit exhibits the typical behaviour when sweeping drive frequency and time (top). When driven on resonance (bottom), the qubit undergoes Rabi oscillations, demonstrating control in the rotating frame.

**Figure 3:**
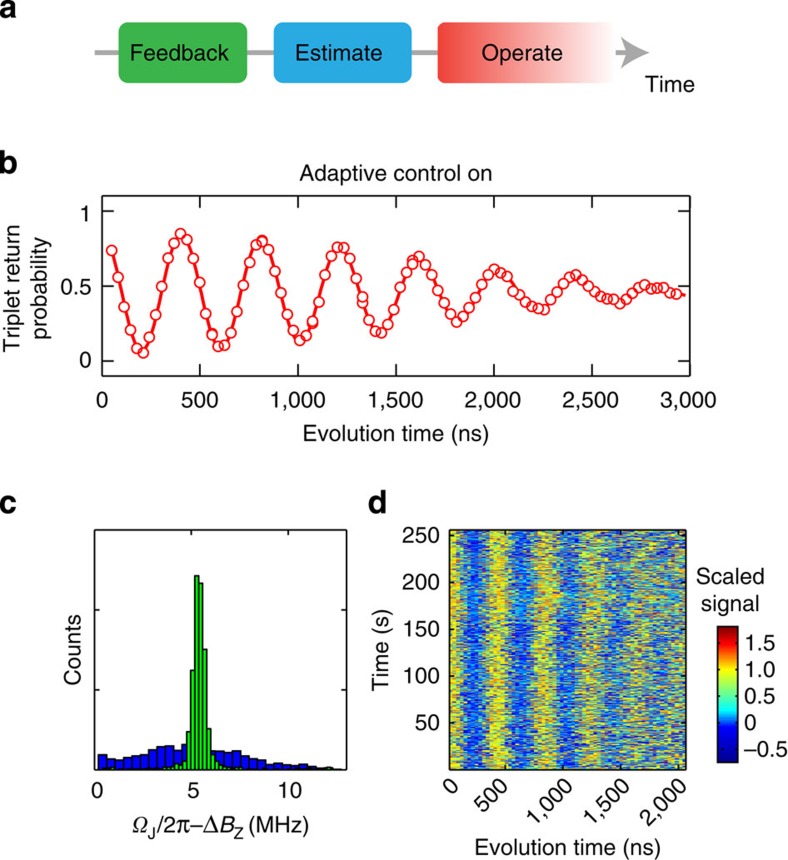
Adaptive control. (**a**) For these measurements we first perform our standard nuclear feedback, then quickly estimate Δ*B*_*z*_ and update the qubit control, then operate the qubit at the correct driving frequency. (**b**) Using adaptive control, we perform a Ramsey experiment (deliberately detuned to see oscillations) and obtain coherence times of 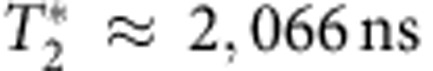. (**c**) Histograms of measured Ramsey detunings with (green) and without (blue) adaptive control. For clarity, these data were taken with a different mean detuning than those in **b**. (**d**) Raw data for 1,024 consecutive Ramsey experiments with adaptive control lasting 250 s in total. A value of 1 corresponds to |*T*_0_› and 0 corresponds to |*S*›. Stabilized oscillations are clearly visible in the data, demonstrating the effect of adaptive control.

We implement this algorithm in real-time on a field-programmable gate array (FPGA), computing *P*(Δ*B*_*z*_) for 256 values of Δ*B*_*z*_ between 50 and 70 MHz. With each measurement *m*_*k*_, the read-out signal is digitized and passed to the FPGA, which computes *P*(Δ*B*_*z*_) and updates an analogue voltage that tunes the frequency of a voltage controlled oscillator (Fig. 1a; Supplementary Note 1, Supplementary Fig. 3). Following the *N*th sample, 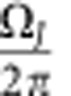 nearly matches Δ*B*_*z*_, and since the nuclear dynamics are slow, the qubit can be operated with long coherence without any additional complexity. To quantify how well the FPGA estimate matches Δ*B*_*z*_, we perform a Ramsey experiment (deliberately detuned to observe oscillations) with this real-time tracking of Δ*B*_*z*_ and find optimal performance for *N*≈120, with a maximum experimental repetition rate, limited by the FPGA, of 250 kHz and a sampling time *t*_samp_=12 ns. Under these conditions, and making a new estimate after every 42 Ramsey experiments, we observe 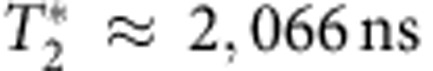, a 30-fold increase in coherence (Fig. 3b). We note that these data are taken with the same pulse sequence as those in Fig. 2d. To further compare qubit operations with and without this technique, we measure Ramsey fringes for ≈250 s (Fig. 3d), and histogram the observed Ramsey detunings. With adaptive control we observe a stark narrowing of the observed frequency distribution, consistent with this improved coherence (Fig. 3c).

## Discussion

**Figure 4:**
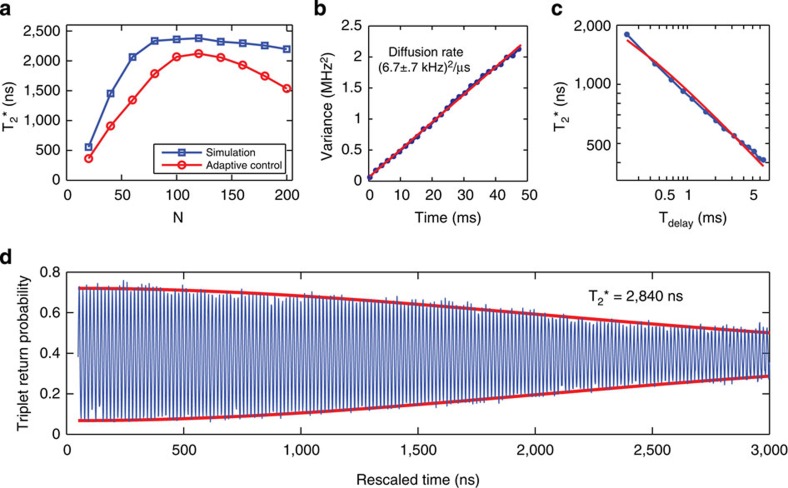
Δ*B*_*z*_ diffusion. (**a**) The coherence time, 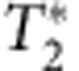 using the adaptive control and for a simulation show a peak, indicating that there is an optimal number of measurements to make when estimating Δ*B*_*z*_. (**b**) When many time traces of Δ*B*_*z*_ are considered, their variance grows linearly with time, indicating a diffusion process. (**c**) The scaling of 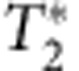 as a function of *T*_delay_ for software scaled data is consistent with diffusion of Δ*B*_*z*_. The red line is a fit to a diffusion model. (**d**) The performance of the Bayesian estimate of Δ*B*_*z*_ can be estimated using software post-processing, giving 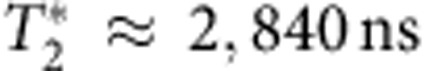, which corresponds to a precision of 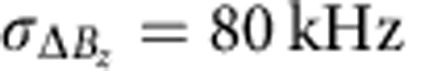.

Although the estimation scheme employed here is theoretically predicted to improve monotonically with *N* (ref. 13), we find that there is an optimum (*N*≈120), after which 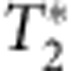 slowly decreases with increasing *N* (Fig. 4a). A possible explanation for this trend is fluctuation of the nuclear gradient during the estimation period. To investigate this, we obtain time records of Δ*B*_*z*_ using the Bayesian estimate and find that its variance increases linearly in time at the rate of (6.7±0.7 kHz)^2^ μs^−1^ (Fig. 4c). The observed linear behaviour suggests a model where the nuclear gradient diffuses, which can arise, for example, from dipolar coupling between adjacent nuclei. Using the measured diffusion of Δ*B*_*z*_, we simulate the performance of the Bayesian estimate as a function of *N* (see Methods). Given that the simulation has no free parameters, we find good agreement with the observed 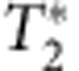, indicating that indeed, diffusion limits the accuracy with which we can measure Δ*B*_*z*_ (Fig. 4a).

This model suggests that increasing the rate of measurements during estimation will improve the accuracy of the Bayesian estimate. Because our FPGA limits the repetition rate of qubit operations to 250 kHz, we demonstrate the effect of faster measurements through software post-processing with the same Bayesian estimate. To do so, we first use the same estimation sequence, but for the operation segment, we measure the outcome after evolving around Δ*B*_*z*_ for a single evolution time, *t*_evo_, rather than performing a rotating frame Ramsey experiment, and we repeat this experiment a total of *N*_tot_ times. In processing, we perform the Bayesian estimate of each Δ*B*_*z,i*_, sort the data by adjusted time 
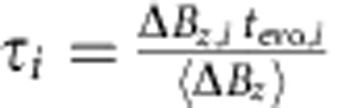
 (for *i*=1,2,…*N*_tot_), and average together points of similar *τ* to observe oscillations (see Methods). We fit the decay of these oscillations to extract 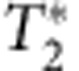 and the precision of the Bayesian estimate, 
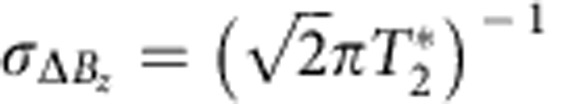
. For the same operation and estimation parameters, we find that 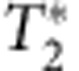 extracted from software post-processing agrees with that extracted from adaptive control Supplementary Fig. 4, Supplementary Note 2. Using a repetition rate as high as 667 kHz, we show coherence times above 2,800 ns, corresponding to an error of *σ*_Δ*Bz*_=80 kHz (Fig. 4d), indicating that improvements are easily attainable by using faster (commercially available) FPGAs.

In addition, we use this post-processing to examine the effect of this technique on the duty cycle of experiments as well as the stability of the Δ*B*_*z*_ estimate. To do so we introduce a delay *T*_delay_ between the estimation of Δ*B*_*z*_ and the single evolution measurement performed in place of the operation. We find 
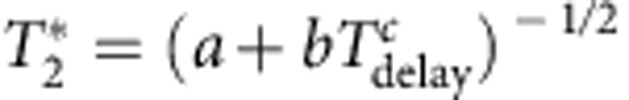
, where c=0.99 (Fig. 4c), consistent with diffusion of Δ*B*_*z*_. Indeed, this dependence underscores the potential of adaptive control, since it demonstrates that after a single estimation sequence, the qubit can be operated for >1 ms with 
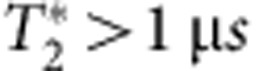
. Thus, adaptive control need not significantly reduce the experimental duty cycle.

In this work, we have used real-time adaptive control on the basis of Hamiltonian parameter estimation of a *S*−*T*_0_ spin qubit to prolong 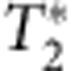 from 70 ns to >2 μs. Dephasing due to nuclear spins has long been considered a significant obstacle to quantum information processing using semiconductor spin qubits^18^, and elimination of nuclear spins is an active and fruitful area of research^19^,^20^,^21^. However, here we have shown that with a combination of nuclear feedback, rotating frame *S*−*T*_0_ spin resonance, and real-time Hamiltonian estimation, we are able to achieve ratios of coherence times to operation times in excess of 200 without recourse to dynamical decoupling^12^,^22^,^23^. If the same adaptive control techniques were applied to gradients as high as 1 GHz (ref. 10), ratios exceeding 4,000 would be possible, and longer coherence times may be attainable with more sophisticated techniques^13^. Though the observed coherence times are still smaller than the Hahn echo time, 
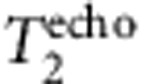
 (ref. 12), the method we have presented is straightforward to implement, compatible with arbitrary qubit operations, and general to all qubits that suffer from non-Markovian noise. Looking ahead, it is likely, therefore, to have a key role in realistic quantum error correction efforts^24^,^25^,^26^,^27^, where even modest improvements in baseline error rate greatly diminish experimental complexity and enhance prospects for a scalable quantum information processing architecture.

## Methods

### Bayesian estimate

We wish to calculate the probability that the nuclear magnetic field gradient has a certain value, Δ*B*_*z*_, given a particular measurement record comprising *N* measurements. We follow the technique in Sergeevich *et al.*^13^ with slight modifications. Writing the outcome of the *k*th measurement as *m*_*k*_, we write this probability distribution as


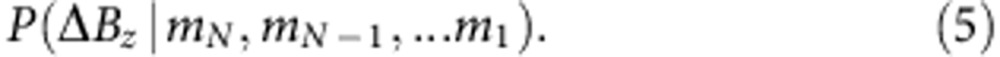


To arrive at an expression for this distribution, we will write down a model for the dynamics of the system, that is, *P*(*m*_*N*_,*m*_*N*−1_,...*m*_1_|Δ*B*_*z*_). Using Bayes’ rule we can relate the two equations as


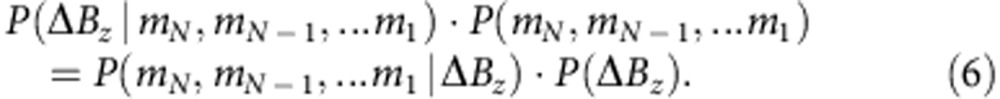


First, we seek a model that can quantify *P*(*m*_*N*_,*m*_*N*−1_,...*m*_1_|Δ*B*_*z*_) that accounts for realistic errors in the system, namely measurement error, imperfect state preparation and error in the axis of rotation around the Bloch sphere. For simplicity, we begin with a model that accounts only for measurement error. Denoting the error associated with measuring a |*S*› (|*T*_0_›) as *η*_S_ (*η*_T_), we write


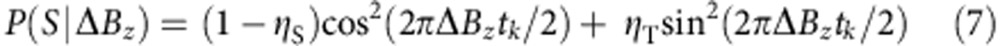



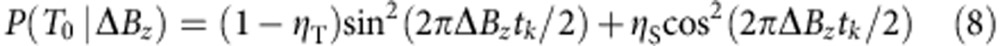


We combine these two equations and write


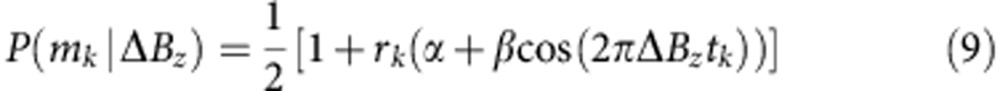


where *r*_*k*_=1 (−1) for *m*_*k*_=|*S*›(|*T*_0_›) and *α* and *β* are given by


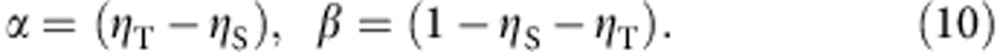


Next, we generalize the model to include the effects of imperfect state preparation, and the presence of non-zero *J* during evolution, which renders the initial state non-orthogonal to the axis of rotation around the Bloch sphere (see above). We assume that the angle of rotation around the Bloch sphere lies somewhere in the *x*–*z* plane and makes an angle *θ* with the *z* axis. We define *δ*=cos^2^ (*θ*). Next, we include imperfect state preparation by writing the density matrix *ρ*_init_=(1−*ε*)|*S*› ‹*S*|)+*ε*|*T*_0_› ‹*T*_0_|. With this in hand, we can write down the model


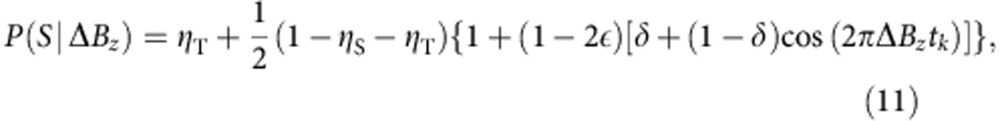



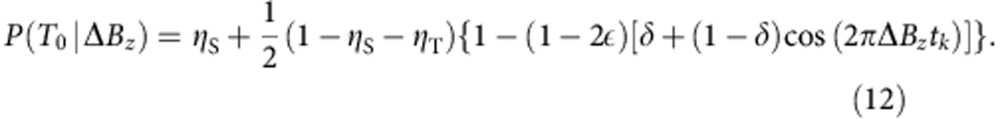


Using the same notation for *r*_*k*_=1 (−1) for *m*_*k*_=|*S*›(|*T*_0_›), we rewrite this in one equation as


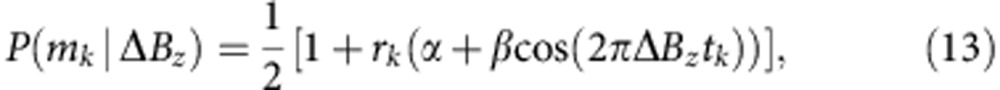


where we now have


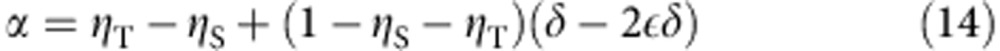



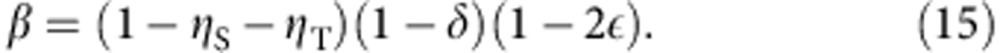


We find the best performance for *α*=0.25 and *β*=0.67, which is consistent with known values for qubit errors.

We next turn our attention to implementing Bayes’ rule to turn this model into a probability distribution for Δ*B*_*z*_. First, we assume that all measurements are statistically independent, allowing us to write


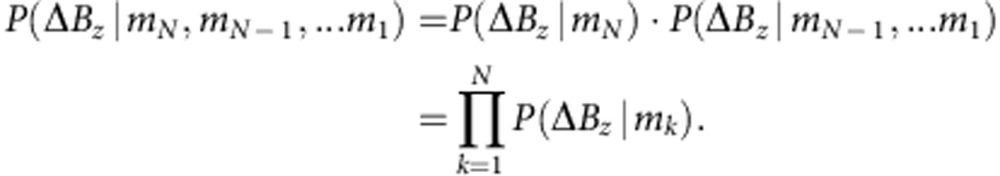


We next use Bayes rule (6) and rewrite this equation as


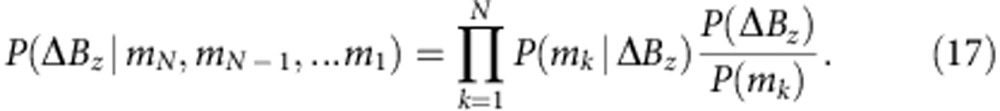


Using our model (13) we can rewrite this as


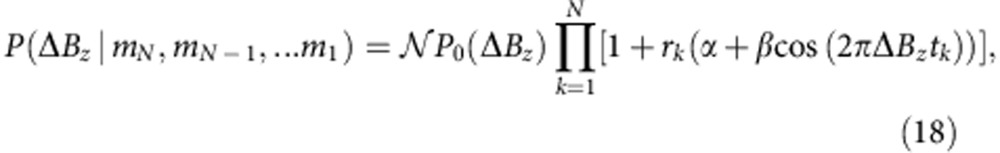


where *N* is a normalization constant, and *P*_0_(Δ*B*_*z*_) is a prior distribution for Δ*B*_*z*_ which we take to be a constant over the estimation bandwidth, and to which the estimator is empirically insensitive. With this formula, it is simple to see that the posterior distribution for Δ*B*_*z*_ can be updated in real time with each successive measurement. After the *N*th measurement, we choose the value for Δ*B*_*z*_, which maximizes the posterior distribution (18).

### Simulation with diffusion

We simulate the performance of our software scaling and hardware (FPGA) estimates of Δ*B*_*z*_ using the measured value of the diffusion rate. We assume that Δ*B*_*z*_ obeys a random walk, but assume that during a single evolution time *t*_*k*_, Δ*B*_*z*_ is static. This assumption is valid when 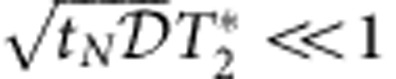, where is the diffusion rate of Δ*B*_*z*_. For an estimation of Δ*B*_*z*_ with *N* different measurements, we generate a random walk of *N* different values for Δ*B*_*z*_ (using the measured diffusion), simulate the outcome of each measurement, and compute the Bayesian estimate of Δ*B*_*z*_ using the simulated outcomes. By repeating this procedure 4,096 times, and using the mean squared error, MSE=‹(Δ*B*_*z*_−Δ*B*_*z*_^estimated^)^2^› as a metric for performance, we can find the optimal number of measurements to perform. To include the entire error budget of the FPGA apparatus, we add to this MSE the error from the phase noise of the VCO, the measured voltage noise on the analogue output controlling the VCO, and the diffusion of Δ*B*_*z*_ during the ‘operation’ period of the experiment.

### Software post-processing

The estimate of Δ*B*_*z*_ can be independently verified using software analysis. In this experiment, we use the same method to estimate Δ*B*_*z*_ as in the adaptive control experiment, but in the operation segment perform oscillations around Δ*B*_*z*_ for verification. We choose *m* different evolution times and measure each *n* times for a total of *N*_tot_=*m* × *n* measurements of Δ*B*_*z*_. In the *i*th experiment (*i*=1,2,…*N*_tot_), we evolve for a time *t*_evo,*i*_, accumulating phase *φ*_*i*_=Δ*B*_*z,i*_*t*_evo*,i*_. Because we make a precise measurement of Δ*B*_*z*_ at the start of each experiment, we can employ it to rescale the time, *t*_evo,*i*_, so that the phase accumulated for a given time is constant using the equation,


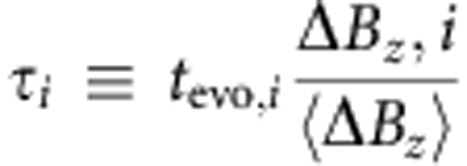


This sets *φ*_*i*_(*τ*_*i*_)=‹Δ*B*_*z*_›*τ*_*i*_, with residual error arising from inaccuracy in the estimate of Δ*B*_*z,i*_. The data are then sorted by *τ*, and points of similar *τ* are averaged using a Gaussian window with *σ*_*τ*_=0.5 ns≪*T*≈16 ns, where T is the period of the oscillations.

## Author contributions

V.U. prepared the crystal M.D.S. fabricated the sample, J.M.N. programmed the FPGA, M.D.S, S.P.H., J.M.N., S.D.B, A.C.D, and A.Y. carried out the experiment, analyzed the data, and wrote the paper.

## Additional information

**How to cite this article**: Shulman, M. D. *et al.* Suppressing qubit dephasing using real-time Hamiltonian estimation. *Nat. Commun.* 5:5156 doi: 10.1038/ncomms6156 (2014).

## Supplementary Material

Supplementary Information Supplementary Figures 1-4 and Supplementary Notes 1-2
