# Supplementary material for: Suppressing qubit dephasing using real-time Hamiltonian estimation
Source: Nat Commun. 2014 Oct 8;5:5156. doi: 10.1038/ncomms6156 (PMC4214408; doi:10.1038/ncomms6156)
Supplement: Supplementary Information — Supplementary Figures 1-4 and Supplementary Notes 1-2 [file ncomms6156-s1.pdf]

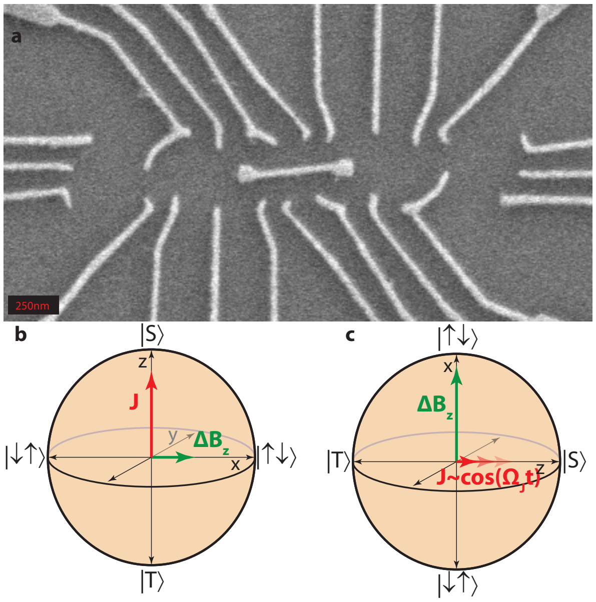

Supplementary Figure 1 | a. An electron microscope image of the device used. Two qubits each comprising a double quantum dot and an additional quantum dot for charge sensing are fabricated in close proximity. A floating metal gate is fabricated between the qubits to increase the inter-qubit capacitance. The right qubit is left inactive for this work. **b**. The Bloch sphere representation for the S-T_0 qubit. **c**. The Bloch sphere representation for the S-T_0 qubit in the rotating frame, where rotations are driven my modulating J.

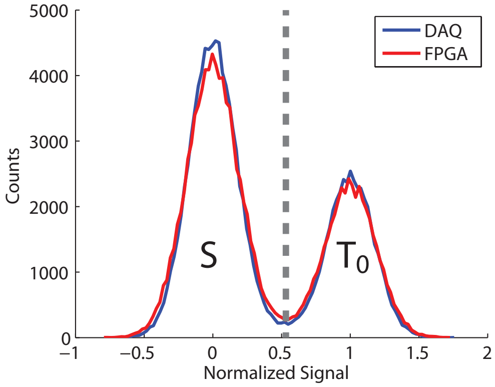

Supplementary Figure 2 | a. A histogram of values measured by the data acquisition card (DAQ) and the FPGA/CDS show nearly identical double peaked structures, indicating that they are capable of consistent singleshot readout. The difference in the heights of the two peaks is caused by residual exchange (J) during evolution, which causes the axis of evolution around the Bloch sphere to be non-orthogonal to the initial state. For the Bayesian estimate, which requires discretized data ($r_k = \pm 1$), we choose a threshold (grey dashed line) corresponding to the minimum between the peaks for the adaptive control on the FPGA.The dashed line is chosen as the threshold for estimating ΔB_z with the FPGA.

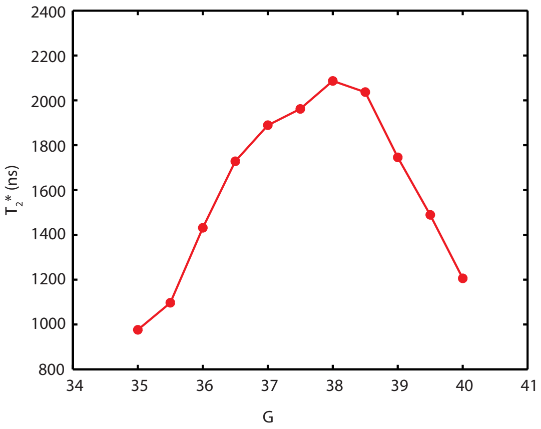

Supplementary Figure 3 | a. T_2^* changes with the gain, G, converting a frequency index into a control voltage for the VCO. This allows for the optimal gain to be found.

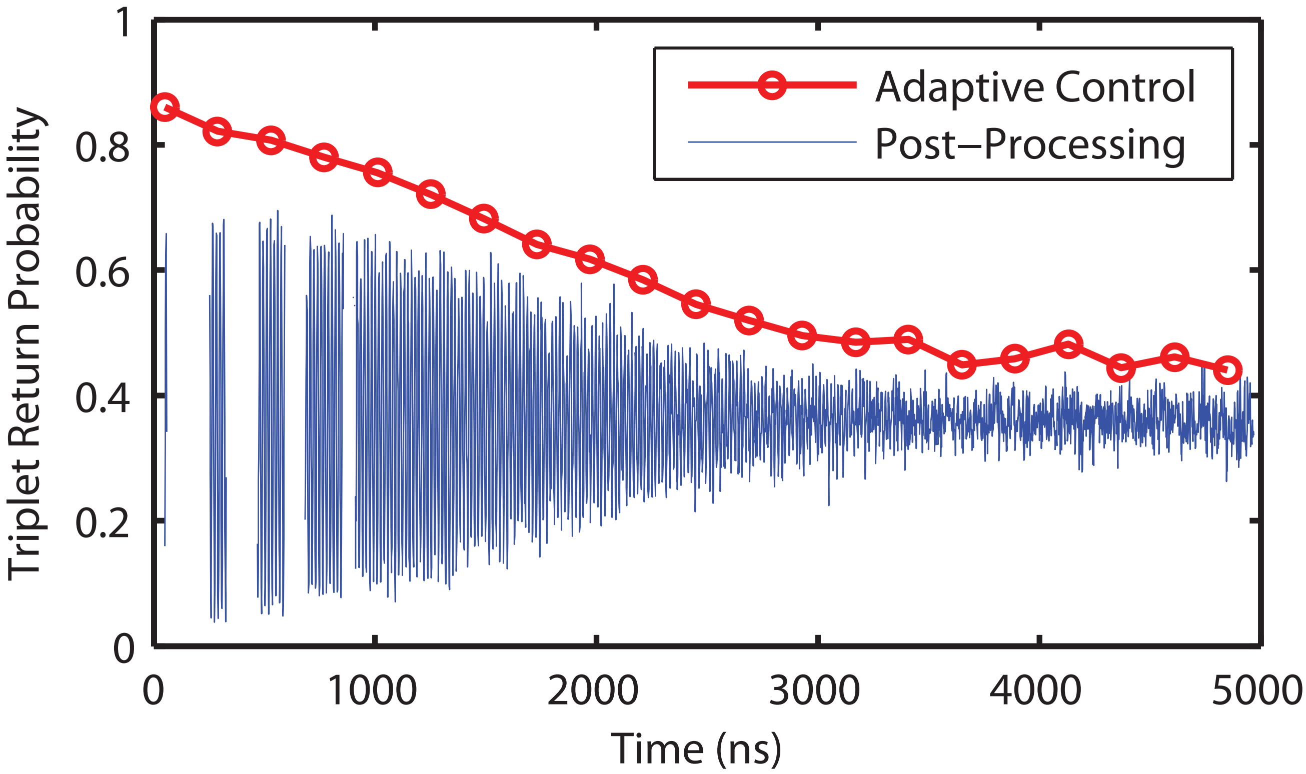

Supplementary Figure 4 | a. When using the same estimation sequence, post-processed oscillations (blue) and data taken using adaptive control (red) show the same decay, indicating similar performance of the estimation. The post-processing technique allows us to explore estimation sequences that are too fast for the FPGA.

Supplementary Note 1 FPGA and experimental apparatus

The reflected readout drive signal returns to room temperature through a cryogenic circulator and amplifier at 4K. The signal is amplified again at room temperature before being demodulated to DC. This DC signal is split and sent to a digitizing card (AlazarTech 660) in a computer and a home built correlated double sampler (CDS). The CDS digitizes the signal and performs a local reference subtraction to reject low frequency noise. The resulting 16 bit signal is converted to a low voltage digital signal and sent to the FPGA for processing. The FPGA is a National Instruments model PXI-7841R and is clocked at 40MHz to maximize processing speed. The probability $P(\Delta B_z|m_k)$ is computed for 256 consecutive frequencies in the estimation bandwidth, $\mathscr{B}$, in two parallel processes on the FPGA to decrease calculation time. Since $\mathscr{B} \approx$ 40MHz is larger than the residual fluctuations of ΔB_z, we increase the frequency resolution by computing the Bayesian estimate of ΔB_z for the the middle 256 frequencies inside of $\mathscr{B}$. For these parameters, the minimum calculation time is 3.7μs for a single t_k. The probability distributions are stored and updated as single-precision floating-point numbers, since we find that single-precision improves the accuracy of the estimator over fixed-point numbers.

After estimating ΔB_z, the FPGA returns the index (an integer between 1 and 256) of the most probable frequency, which must be converted to a voltage to control the VCO. To do so, we apply a linear transformation to the index, $V = G \times index + O$, where the O controls the detuning of the driving frequency. We tune the G to maximize T_2^* using adaptive control (Figure 3**a**).

Supplementary Note 2 Software Post Processing

To compare post-processing with adaptive control, we first perform the same estimation sequence for both software post-processing and adaptive control, with a 250 kHz repetition rate, t_{samp} = 12 ns and N =120, followed by an operation sequence of 30 measurements. We find T_2^* = 2148 ± 30 ns with software and T_2^* = 2066 ns with adaptive control, showing good agreement between the two approaches (Figure 4**a**).

For the software post-processing, we can reduce the amount of diffusion that occurs during the operation sequence by performing only one verification measurement following the same estimation sequence, enhancing T_2^*, to 2580 ± 40 ns. For the software rescaling in Fig. 4d, the 109 estimations were performed in 225 μs instead of the 440 μs used by the FPGA, yielding T_2^* = 2840 ± 30 ns. This is likely limited by diffusion and the precision of the estimator with N=109.
